# Thymoquinone Augments Cyclophosphamide-Mediated Inhibition of Cell Proliferation in Breast Cancer Cells

**DOI:** 10.31557/APJCP.2019.20.4.1153

**Published:** 2019

**Authors:** Arif Khan, Yousef H Aldebasi, Sultan A Alsuhaibani, Masood A Khan

**Affiliations:** *College of Applied Medical Sciences, Qassim University, Buraidah, Al-Qassim, Saudi Arabia.*

**Keywords:** Thymoquinone (TQ), FASN, Her2, Cyclophosphamide (Cyclo)

## Abstract

**Objective:**

Cancer chemotherapy at the recommended doses is largely associated with toxicity, and also it is not effective enough to reduce the advancement of the disease at lower doses. Thymoquinone (TQ) is an active compound derived from black seeds (*Nigella sativa*) which exhibits anticancer activities. The aim of the present study was to investigate the synergistic effect of TQ alone and in combination with cyclophosphamide (cyclo), and to unravel the role of TQ in fatty acid synthase (FASN) mediated molecular signaling in Her2 + and Her2- breast cancer cell lines.

**Methods:**

The effect of TQ on the growth of Her2+ SKBR-3 and Her2- MDA-231 breast cancer lines were evaluated as percent cell viability by cytotoxicity-based MTT assay. The analysis of cell cycle arrest was done through flow-cytometry followed by Western blot and RT-PCR to detect signaling events in the cells.

**Results:**

The data showed that TQ-cyclo (0.5mM-10µM) combination significantly inhibited the proliferation through the 5.49% and 57.72% accumulation of cells in sub-G1 and G1 respectively as 12% cells were shifted from G2/M phase in Her2+ breast cancer cells. Similarly, TQ-cyclo (0.5mM-20µM) combination exhibited that the 16.6% cells were arrested in Sub-G1 and only 3.54% cells were remained in G2/M phase as it was 22.89% in DMSO control in Her-2- breast cancers cells. Though TQ alone or in combination with cyclo alleviated the PI3K/Akt signaling by downregulating the phosphorylation of Akt and upregulating the PTEN, no changes was observed in FASN and Her-2 as well in both type of cells. The significant decreased expression of cyclin D1 was found in TQ-cyclo combinations.

**Conclusion:**

The current findings suggested that TQ can alter the cell cycle progression and induce cell death independent of FASN mediated signaling. In terms of clinical perspective, the present study clearly showed that TQ can broadly augment the effect of cyclo in breast cancer cases irrespective of Her-2+ or Her-.

## Introduction

Anticancer drugs are usually afflicted with toxic manifestations at required amount of doses to control the process of carcinogenesis at various stages. In recent years, the potential of active dietary constituents to fight against the cancer has engrossed widespread attention. Thymoquinone (TQ) is the main bioactive constituent of black seeds (*Nigella sativa*) which has been shown to possess many biological activities relevant to human cancer prevention and treatment. As substantiated from several studies, TQ is a promising anti-cancer therapeutic compound affecting multiple signaling pathways that can control cell division and growth, apoptosis, inflammation, angiogenesis, and metastasis (Reddy et al., 2003; Yi et al., 2008; Arafa et al., 2011; Younus., 2018; Rajput et al., 2013). The therapeutic implications of liposomal TQ to combat fluconazole-resistant *Candida albicans* in a murine model has been proved earlier (Khan et al., 2015). Combining the active chemotherapeutic reagent at the lowest level of toxicity with agents that target specific molecular mechanism offers a promising strategy for the treatment of cancer and may counteract human cancer cells resistant to the drugs (Norwood et al., 2007; Lei et al., 2012; Williams et al., 2014; Harpole et al., 2015).

Several studies have suggested fatty acid synthase (FASN) as a potential molecular target in the treatment of cancers, especially breast cancer (Rysman et al., 2010; Ventura et al., 2015; Gonzalez-Guerrico et al., 2016). As evident from human studies, FASN is upregulated in infiltrating carcinomas in comparison to non-transformed epithelial tissues adjacent to tumors (Kuhaida et al., 2000; Bhatt et al., 2012; Cai et al., 2014). Moreover, it is eminent that higher level of FASN may regulate oncogenic proteins associated with malignant transformation, suggesting its important role in the progression of cancer. Interestingly, many studies have shown the interplay between FASN and cellular localization of Her-2 oncogene in breast cancer cells. Therefore, substantial interest has been targeted toward identifying natural occurring active dietary constituents as inhibitors of this enzyme. The potential of neutraceuticals such as green tea polyphenol epigallocatechin-3-gallate (EGCG) and other flavonoids against breast cancer through downregulating the FASN activity has been suggested by previous studies (Pan et al., 2007; Puig et al., 2008; Khan et al., 2014). We previously reported the correlation of resveratrol-induced inhibition of cell proliferation with the status of FASN and Her2 expression in breast cancer cells. We found that resveratrol constrained the growth of Her2 expressed breast cancer cells by inhibiting FASN in a dose dependent manner. Keeping these views into considerations, the aims of the present study were to analyze the effect of TQ alone or in combination with low dose of cyclophosphamide , and to determine the role of FASN in TQ mediated growth inhibition.

## Materials and Methods

*Materials*

Cyclophosphamide Thymoquinone and polyvinylidene fluoride (PVDF) membrane were purchased from Santa Cruz Biotechnology, Inc. (California, USA). Antibodies (Abs) against p185Her2, FASN, Cyclin D1, β-actin, PEA3, Akt, phospho-Akt (Ser473), p85-PI3K, PTEN, and Rabbit anti mouse horseradish peroxidase or goat anti rabbit horseradish peroxidase-conjugated secondary antibodies were purchased from ABCAM (Cambridge, USA). The polyvinylidene fluoride (PVDF) membrane was obtained from Santa Cruz Biotechnology. The rest of the chemicals were of analytical grade of purity and were procured locally.

*Cell culture*

SKBR-3 and MDA-231 breast cancer cells were procured from American Type Culture Collection (ATCC), and sub-cultured in DMEM from Santa Cruz Biotechnology Inc. (California, USA) comprising of 10% fetal bovine serum (FBS), 1% L-glutamine, 1% sodium pyruvate, 50 U/ml penicillin, and 50 mg/ml streptomycin from Sigma-Aldrich (Missouri, USA). The culture conditions of the cells were maintained at 37^o^C in a humidified atmosphere of 95% air and 5% CO_2_.

*Treatment of cells*

**Figure 1:**
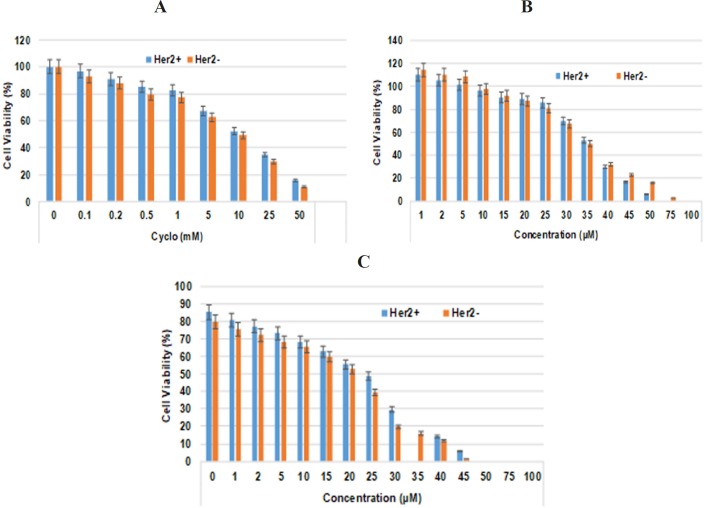
Effect of TQ and Cyclo on Her2+ and Her2- Breast Cancer Cells. (A) Varying concentrations of cyclo (B) Varying concentrations of TQ (C) Varying concentration of TQ in combination with constant concentration of cyclo (0.5mM). 1 X 10^4^ cells (200 µl) were treated with different concentrations of TQ (0, 1, 2, 5, 10, 15, 20, 25, 30, 35, 40, 45, 50, 75 and 100 µM) for 48 h. The percentage of cell growth inhibition was measured by cell cytotoxicity assay as described in methods section. Values are represented as the percentage of viable cells; data represent mean percentages of viable cells ± SD of three independent experiments

The primary screening was performed to evaluate the effects of cyclophosphamide on the inhibition of cell proliferation at different concentrations, including 0.1, 0.2, 0.5, 1.0, 2.5, 5.0 10.0, 25.0, and 50.0 mM by MTT cell proliferation assay. According to the inhibition of cell proliferation, 0.5mM dose was selected which showed only 15-20 % cell death. The stock solution of TQ (1 mM) was prepared in dimethyl sulphoxide (DMSO) and diluted with complete medium (without antibiotics) to achieve the required concentration used for the treatment of cells (Figure 1a). The cells were exposed to different concentrations of TQ ( 0, 1, 2, 5, 10, 15, 20, 25, 30, 35, 40, 45, 50, 75 and 100 µM) for 48 h. In the controls, the cells were incubated with the vehicle (DMSO) alone.

*Cell cytotoxicity assay*

The cell cytotoxicity assay kit (colorimetric) from abcam was used to assess the cell viability according to manufacturer’s instructions. Briefly, 1 X 10^4^ cells (200 µl) were plated in 96-well microtitre plates in culture medium followed by the treatment of cells with various concentrations of TQ and incubated at 37^o^C in CO_2 _incubator. Following 48 h of incubation with TQ alone and TQ in combination with cyclo (0.5mM), reagent was added, and absorbance of the samples were taken at 570 nm and 605 nm on a multiwall plate reader. The ratio of OD570 to OD605 was used to determine the cell viability in each well. The percentage of cell viability was calculated using the following formula:

% Cell viability = 100 X (R_sample_ – R_o_) / (R_ctrl_ – R_o_)

R_sample_ is the absorbance ratio of OD_570_/OD_605_

R_ctrl_ is the absorbance ratio of OD_570_/OD_605_ DMSO control

R_o_ is the average background (non-cell control) absorbance ratio of OD_570_/OD_605_

*Flow-cytometric analysis of cell cycle*

1 X 10^6^ cells grown in 100-mm culture plates were treated with two selected doses of TQ (0, 10, 20 mM) for 48 h after. The treated cells were harvested in phosphate-buffered- saline (PBS) followed by fixation in 70 % ice-cold ethanol. After incubation for 24 h, cells were treated with 50 µg/ml propidium iodide (PI) and 100 µg/ml Ribonuclease A (RNAse A) and incubated at 37^o^C for 30 min. The samples were measured using MACSQuant Analyzer 10 and raw data were acquired and analyzed for flow cytometry using MACSQuantify software version 2.4 (Miltenyi biotec, Germany).

**Figure 2:**
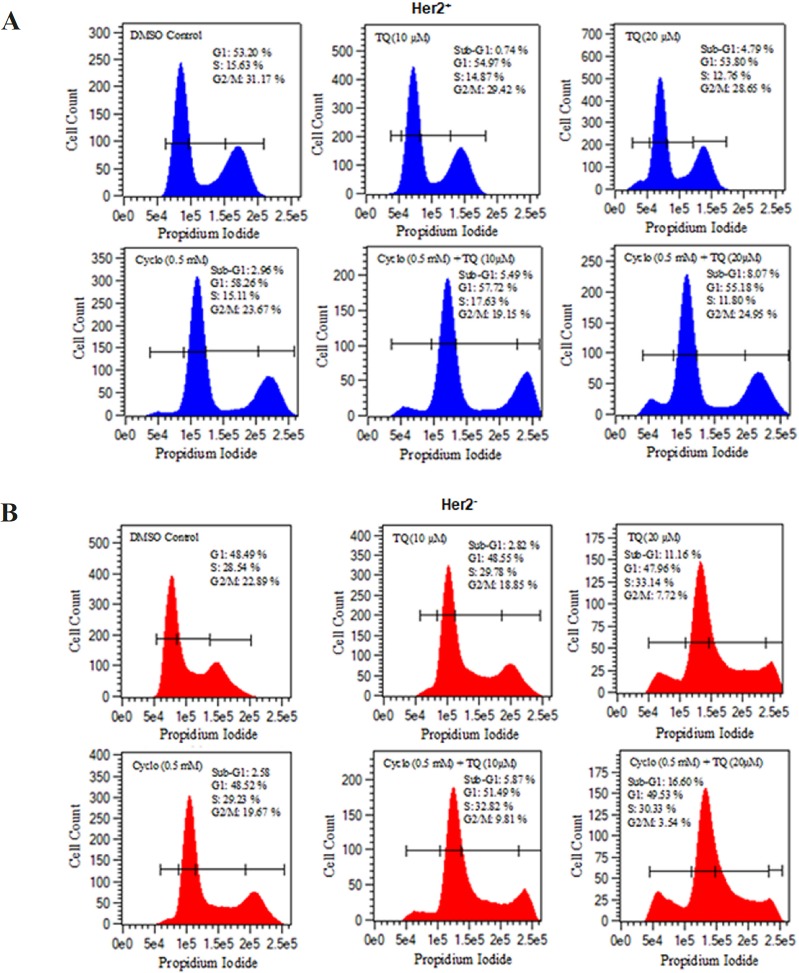
Effect of TQ, Cyclo and TQ-Cyclo Combination on Cell Cycle Progression in (A) HER2+ breast cancer cells (B) Her2- breast cancer cells. 1 x 104 cells (200 µl) were treated with TQ (10, 20 µM) with or without cyclo (0.5mM) for 48 h, and DNA cell-cycle analysis was performed as described in Section 2 . Vehicle control cells showed no sub-G1 peak, whereas TQ-treated cells contained cells in sub-G1, as determined by flow-cytometry analysis; PI fluorescence intensity was measured as an indicator of cellular DNA content

**Figure 3:**
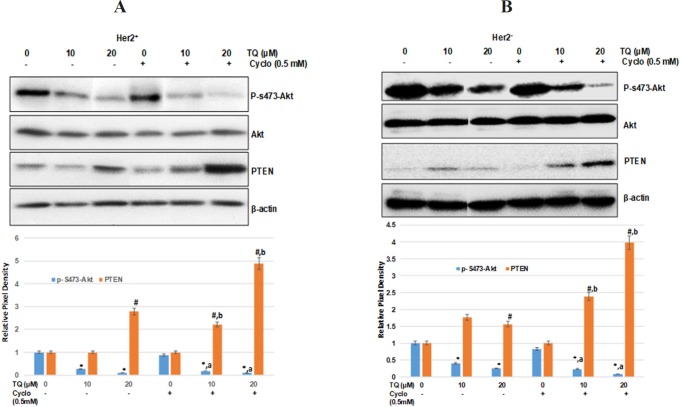
TQ Inhibited Akt Phosphorylation and Increased the Expression of Its Inhibitor PTEN in Dose-Dependent Manner in (A) HER2+ breast cancer cells (B) Her2- breast cancer cells. 1 X 106 cell in 100 mm plates were treated with TQ (10, 20 µM) with or without cyclo (0.5mM) for 48 h. The total protein was isolated and Western blot analysis was performed as described in materials and methods with antibodies specific for phosphorylated-S473-Akt, Akt and PTEN. Data shown are the results of three independent experiments, and were represented as the relative densities of protein bands normalized to ß-actin. *Significant difference compared with vehicle control (0.0 µM) for p-5473-Akt (p<0.001), a Significant difference compared with cyclo alone (0.5 mM) for p-5473-Akt (p<0.001) in Her2+ cells. #Significant difference compared with vehicle control (0.0 µM) for PTEN (p<0.001), #Significant difference compared with cyclo alone (0.5 mM) for PTEN (p<0.001) in Her2- cells

**Figure 4:**
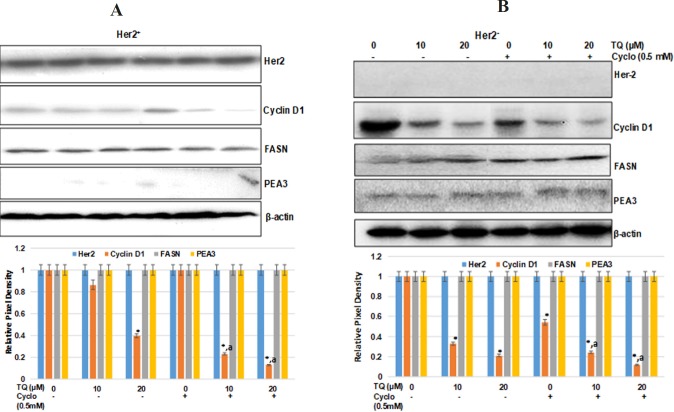
TQ Downregulated the Cyclin-D1 Independent of FASN and Her2 (a) HER2+ Breast Cancer Cells (b) Her2- breast cancer cells. 1 X 106 cell in 100 mm plates were treated with TQ (10, 20 µM) with or without cyclo (0.5mM) for 48 h. The total protein was isolated, and western blot analysis was performed as described in materials and methods section with antibodies specific for p185Her2, Cyclin-D1, FASN, PEA3. Equal loading was confirmed by re-probing the membrane with ß-actin. Data shown are the results of three independent experiments, and were represented as the relative densities of bands normalized to ß-actin. *Significant difference compared with vehicle control (0µM) for Cyclin D (p < 0.001), a Significant difference compared with cyclo (0.5 mM) for Cyclin D (p < 0.001)

**Figure 5:**
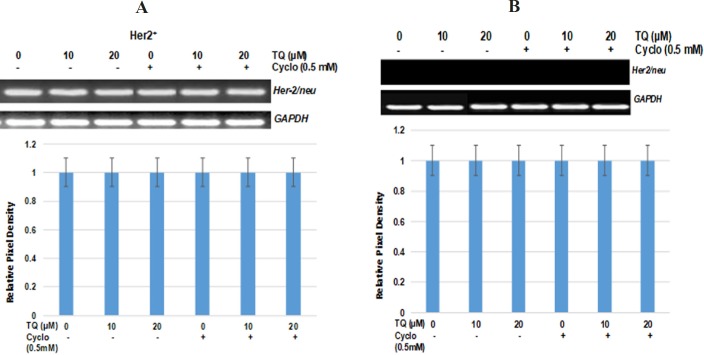
TQ Had Vo Effect on Her2 Promoter Activity Through Binding of Its Transcriptional Regulator PEA3. 1 X 106 cell in 100 mm plates were treated with TQ (10, 20 µM) with or without cyclo (0.5mM) for 48 h. RT-PCR analyses for Her2 and GADPH transcripts and expression were performed as described in materials and methods section. Data shown are the results of three independent experiments and were represented as the relative densities of bands normalized to GAPDH

*Western-blot analysis*

Western blotting was carried out as described previously (Chou et al., 1984). Briefly, BCA protein assay kit (Thermo Scientific, Rockford, USA) was used to estimate the concentration of protein in cell lysates. Proteins (40 mg) were resolved on 6-12% sodium dodecyl sulphate (SDS)-polyacrylamide gels and transferred on PVDF membranes in wet protein transfer system. The blots were blocked for 30-40 minutes with 5% skimmed milk followed by probing with required antibodies at dilutions instructed by the suppliers. The detection of immunoblots through chemiluminescence was done using enhanced chemiluminescence reagents obtained from Millipore (Billerica, USA). Data were presented as the relative density of protein bands normalized to b-actin as membranes were re-probed with it as an internal control. The intensities of the bands were measured using Image Analysis software on an Image Gel Documentation System.

*Reverse transcriptase-polymerase chain reaction (RT-PCR) assay*

Total RNA was extracted using MagaZorb® Total RNA Mini-Prep Kit (promega) followed by RT-PCR for Her-2 transcript on Bio-Rad C1000 Touch Thermal Cycler by using reverse transcription system (Promega) according to the manufacturer’s instructions. The primers sequences to amplify Her-2 transcript were 50-GACCCGCTGAACAATACCAC-30 (forward) and 50-TGCCGTCGTCTTCTAGGCCTTCAT-30 (reverse). In each reaction, GAPDH cDNA was amplified as an internal. Reaction mixture was first denatured at 95^o^C for 10 min. PCR conditions were 95^o^C for 1 min, 60^o^C for 2 min, and 72 8^o^C for 2 min for 30 cycles followed by 72^o^C for 10 min.

*Statistical analysis*

Means and standard deviation (SD) were calculated for all treated samples and vehicle controls. The Student’s t-test for the paired samples and one way ANOVA Holm-Sidak for different treated sample were used to evaluate the statistical significance of differences between parameters (Sigma Stat 3.5, Systate Software Inc., San Jose, USA).

## Results

As depicted in Figure 1a, it was found that 0.5 mM concentration of cyclo inhibited the proliferation of cells by ~15 % and 10 % in Her2+ and Her2- breast cancer cells, respectively. which was selected for further studies. 

*TQ amplified the growth inhibition of both Her2+ and Her2- breast cancer cells*

The preliminary screening of TQ was executed to assess the effect of various concentrations of TQ on the cellular proliferation and cell viability using the cell cytotoxicity assay kit. As depicted in Figure 1b and 1c, the incessant degenerations in the viability of Her2+ and Her2- cells were measured with increasing doses of TQ (5–150 µM). The IC_50_ value for growth inhibition was obtained at ~34 mM but reduced to 24 and 22.5 mM in Her2+ and Her2- cells respectively while treated the in combination with 0.5 mM cyclo. Based on these observations, we selected two concentrations of TQ below IC50 of combination i.e. 10 and 20 mM for further mechanistic studies over a 48 h period with constant dose (0.5 mM) of cyclo. 

*TQ enlarged the accumulation of cyclo treated cells in G1 and sub-G1 in both Her2+ and Her- breast cancer cells*

The treatment of cells with TQ in combination with cyclo exhibited that the cells accumulated in sub-G0 and G1 in dose dependent manner. The marginal shift from G2/M and S phase to sub-G1 and G1 was observed in Her2+ cells treated with TQ, while approximately 7 % change from G2/m to G1 was observed in the cells treated with cyclo. Remarkably, very significant transformation was discovered in the cells exposed to TQ in combination with cyclo as 5.49 % cells were arrested in sub-G1 and 57.72 % in G1 as 10 % cells were shifted from G2/M. In the cells treated with TQ 20 µM and Cyclo, showed significant shift into sub-G1 8.07 % from G1 in comparison to control other treated groups (Figure 2a).

In Her2- cells, the accumulation of cells in sub-G1 and G1 were observed dose dependently more significantly in comparison to Her2+ cells. It showed that 11.16 % in sub-G1 and ~48 % cells were arrested while the treated with 20 µM TQ . However, this swing was more prominent in the cells treated with 20µM TQ and cyffclo as only 3.54 % cells were remained in G2/M phase, while 16.6 % cells were recorded in sub-G1 phase (Figure 2b).

*TQ induced the inhibition of Akt phosphorylation alters Akt/PI3K/mTOR pathway*

PI3K kinase regulates the key molecular pathways crucial for biological processes like cell proliferation and cell motility (Chan et al., 1999). The phosphorylation of Akt at S473 plays a vital role in essential cellular by phosphorylating several downstream substrates. Several studies have shown the association between PI3K/AKT signaling pathway and TQ-mediated inhibition of different cell types growth (Khan et al., 2017; Su et al., 2016; Dirican et al., 2015). As shown in Figure 3a and b, simultaneous downregulation and upregulation of pAkt and PTEN were noticed in dose dependently manner, and showed most effective with concomitant exposure of TQ (20 mM) and cyclo (p < 0.001) against vehicle control and cyclo alone as well.

To characterize the possible effect of TQ on Her-2 over-expressed breast cancer cells, we determined the changes in the expression of FASN and Her2/neu in Her2+ and Her2- breast cancer cells. Interestingly, we observed no changes in FASN and Her2 expression in comparison to vehicle in any of the treated groups. These data ruled out the inhibition of cell proliferation by TQ either alone or in combination through FASN mediated signaling . To gain additional insight and confirm the findings, we examined the expression of Her2 regulators PEA3 and Cyclin D1. It is indicated that her-2 promoter activity is suppressed by Ets transcription factor PEA3, leading to inhibition of Her2 mediated carcinogenesis (Oliver et al., 1998; Wang et al., 2000). In this study, no change was observed in PEA3 expression in all the treated groups, but significantly decreased expression of cyclin D1 was recorded in both TQ-cyclo combinations (Figure 4a and b). Additionally, we also tried to examine any transcriptional changes in the expression of Her2 gene other than PEA3. As shown in Figure 4a and b, no change was observed in any of the treated groups in comparison vehicle controls in both of the cells. According to findings of this study, it seems that TQ either alone or in combination with cyclo has no role in Her-FASN mediated signaling.

## Discussion

Given the toxic effect of anticancer conventional drugs at recommended doses, concomitant use of dietary constituents with less amount of synthetic drugs has been suggested. In the last two decades, many naturally occurring substances have been reported to prevent the occurrence of carcinogenesis at various stages (Pitchaiah et al., 2017; Lefranc et al., 2017). In this regard, TQ has shown its huge potential to protect against experimental carcinogenesis and molecular mechanism in various cancer cell lines and different model animals of cancer (Rajput et al., 2013; Salim et al., 2013; Dastjerdi et al., 2016; Khan et al., 2017).

As evident from several studies, the increased activity of FASN in various human cancers, including breast cancer is associated with the progression of the malignant phenotype (Alo et al., 2001; Wang et al., 2004; Fiorentino et al., 2008; Migita et al., 2009; Gonzalez-Guerrico et al., 2016). Therefore, pharmacological inhibitors of FASN have attracted great attention as the suppression of FASN evidently inhibits the cell proliferation and escalates the rate of apoptosis all together in human and mouse melanoma cells (Liu et al., 2010; Benjamin et al., 2015). The potential of nutraceuticals such as green tea polyphenol epigallocatechin-3-gallate (EGCG) and other flavonoids against breast cancer by downregulating the FASN activity has been suggested by previous studies (Tian et al., 2006; Pan et al., 2007; Puig et al., 2008; Khan et al., 2014).

In the present study, our results clearly demonstrated the potential of TQ to amplify the effect of cyclo at a very low dose (0.5 mM). We also identified no role for FASN and Her2 mediated signaling in TQ alone or TQ in combination with cyclo. In the current study, we found that TQ inhibited the cell proliferation independent of any changes in the expression of Her2 either at transcriptional or translation level (Figure 4,5). As depicted in Figure 4a and b, no change was either observed in PEA3, which specifically reverses the in vitro transformed phenotype of Her2+ breast cancer cells by reducing Her2 oncogene promoter activity (Xing et al., 2000).

The current findings suggested that TQ can alter the cell cycle progression and induce cell death independent of FASN mediated signaling. Future studies are suggested to investigate the exact molecular mechanism in the inhibition of cancer cell proliferation for TQ alone and TQ in combination with cyclo. It also paves us the way to prepare the liposome-based various formulations of TQ-cyclo for effective delivery with increasing doses. 

The present study limited to only two types of cells, which need to include several types of breast cancer cells to understand the possible mechanisms of TQ-cyclo combination in detail for further investigations. In terms of clinical perspective, the present study clearly showed that TQ may broadly augment the effect of cyclophosphamide in breast cancer cases irrespective of Her2+ or Her-.

## Funding Statement

This study was supported in part by the Deanship Scientific Research, Grant # 3155, Qassim University, Saudi Arabia.

## Statement conflict of Interest

 The authors declare no competing financial interests.
